# Hospitalization linked to antiepileptics, sedative-hypnotics and anti-Parkinsonian drug poisoning, adverse effects, and underdosing in Australia: An ecological study

**DOI:** 10.1097/MD.0000000000046577

**Published:** 2025-12-12

**Authors:** Abdallah Y. Naser, Hassan Alwafi, Alaa A. Alsharif, Ahmed M. Al Rajeh, Jaber S. Alqahtani, Abdulelah M. Aldhahir, Anan S. Jarab, Yosra J. Alhartani, Asaleh El-Qasem

**Affiliations:** a Department of Applied Pharmaceutical Sciences and Clinical Pharmacy, Faculty of Pharmacy, Isra University, Amman, Jordan; b Department of Clinical Pharmacology and Toxicology, Faculty of Medicine, Umm Al-Qura University, Makkah, Saudi Arabia; c Department of Pharmacy Practice, College of Pharmacy, Princess Nourah bint Abdulrahman University, Riyadh, Saudi Arabia; d Department of Respiratory Care, College of Applied Medical Sciences, King Faisal University, Al-Ahsa, Saudi Arabia; e Department of Respiratory Care, Prince Sultan Military College of Health Sciences, Dammam, Saudi Arabia; f Department of Nursing, Respiratory Therapy Program, College of Nursing and Health Sciences, Jazan University, Jazan, Saudi Arabia; g Health Research Center, Jazan University, Jazan, Saudi Arabia; h Department of Pharmacology and Therapeutics, College of Medicine and Health Sciences, United Arab Emirates University, Al Ain, United Arab Emirates; i Department of Clinical Pharmacy, Faculty of Pharmacy, Jordan University of Science and Technology, Irbid, Jordan; j Faculty of Pharmacy, University of Jordan, Amman, Jordan.

**Keywords:** adverse effects, antiepileptics, antiparkinsonian, poisoning, sedative-hypnotics, underdosing

## Abstract

Poisoning from medications such as antiepileptics, sedative-hypnotics and anti-Parkinsonian drugs remains a major public health concern. These medications contribute significantly to death and illness from intentional or accidental overdose. In Australia, these medications are associated with high rates of hospital admissions. Our study aimed to analyze trends in hospital admissions related to poisoning from antiepileptics, sedative-hypnotics and anti-Parkinsonian drugs in Australia for 24 years, starting from 1998. We collected hospital admission data for poisoning by antiepileptic, sedative-hypnotic and anti-Parkinsonian drugs from the National Hospital Disease Database. Population data were obtained from the Australian Bureau of Statistics. We analyzed based on total trends, type of admission, age, gender, and medication category. A total of 202,705 hospital admission episodes due to poisoning by antiepileptic, sedative-hypnotic and anti-Parkinsonian drugs were recorded in Australia during the study time, with a decreased hospital admissions number and hospital rate of 41.7% and 57.8%, respectively. Overnight-stay admissions accounted for 69.4% of the total admissions. The primary cause of hospital admissions was poisoning by benzodiazepines. Admissions were highest among the age groups 20 to 59 and females. The study demonstrated a substantial decline in hospital admissions for poisoning by antiepileptic, sedative-hypnotic and anti-Parkinsonian drugs in Australia. Still, hospital admission rates increased for certain drug classes. Some population groups were at higher risk of hospital admission. These findings suggest the need for continued monitoring, targeted interventions, and increased awareness among the population to reduce drug poisoning and related hospital admissions.

## 1. Introduction

Poisoning is defined as the contact, ingestion, injection, or inhalation of lethal or harmful gases, toxins, chemicals, or drugs.^[1]^ Globally, it remains a rising central public health concern.^[2–7]^ Unintentional or intentional drug overdosing or poisoning is a significant cause of mortality and morbidity at a global level.^[8]^ In the past decade, many countries have experienced a rapid increase in deaths from drug overdoses.^[9]^ Further, poisoning has resulted in significantly increased mortality rates, long-term hospitalization, repeated emergency room visits, and, therefore, has societal effects.^[2–4]^

Like many other countries, there is an annual increase in the number of drug overdose-related deaths in Australia, with over 35,000 such deaths having been reported since 2001.^[10]^ In 2021, antiepileptic, sedative-hypnotic, and anti-Parkinsonian drugs ranked second among drug classes identified in drug-induced deaths in Australia, contributing to 54% of these deaths.^[11]^ During the same period, among individuals aged 15 to 24, 65 to 74, and 75 years and over, the drugs that resulted in the highest mortality rates were antiepileptic, sedative-hypnotic and anti-Parkinsonian drugs.^[11]^

Severe challenges arise for anesthesiologists, medical intensivists, and emergency medicine staff due to sedative-hypnotic drug-related acute poisoning, which frequently causes hypoventilation.^[12]^ Patients may be at increased risk for drug use disorders or intoxication due to treatment, which may require medications such as antiepileptics, antidepressants, and anxiolytics to treat symptoms of pain, and psychiatric and neurological disorders.^[13,14]^ Additionally, among patients treated in toxicology departments globally, it is common for antiepileptic drugs to emerge as a significant reason for acute poisoning due to their extensive utilization.^[15]^

During 2020 to 2021 in Australia, 40% of intentional self-harm-related hospitalizations were attributed to psychotropic, anti-Parkinsonian, sedative-hypnotic and antiepileptic drugs, which was the most common type of self-harm resulting in hospitalizations between 2008–2009 and 2020–2021.^[16]^ During 2021 to 2022 in Australia, 6.1% (8200 hospital admissions) of drug-related hospital admissions were due to antiepileptic, sedative-hypnotic and anti-Parkinsonian drugs,^[17]^ with benzodiazepines accounting for some 46% of these hospital admissions cases. Despite these existing trends, there remains a gap in understanding them; thus, there is an urgent need for targeted intervention strategies. Therefore, this study aims to examine trends in hospital admissions associated with poisoning by Parkinsonian these drugs in Australia, with a focus on demographic characteristics, types, and rationales for hospital admission.

## 2. Methods

### 2.1. Data sources

#### 2.1.1. National Hospital Morbidity Database

The National Hospital Disease Database is maintained by the Australian Institute of Health and Welfare and contains data from public and private hospitals in Australia.^[18]^ It includes information on hospital procedures, length of stay, diagnoses, and patient demographics. The purpose of the database is to collect data on the medical care provided to patients admitted to Australian hospitals.^[19]^

#### 2.1.2. Australian Bureau of Statistics (ABS)

We used the ABS [the nation’s official statistical agency] to collect mid-year population data between 1998 and 2022; historical population data^[20]^ to extract data between 1998 and 2016, while national, state, and territory population data^[21]^ were used to extract data between 2017 and 2022. The ABS produces independent and dependable data.^[22]^

### 2.2. Study population

The study encompassed all hospital admissions related to poisoning by antiepileptic, sedative-hypnotic and anti-Parkinsonian drugs in Australia from 1998 to 2022. That includes all available data from National Hospital Disease Database principal diagnosis data regarding hospital admissions for poisoning by Parkinsonian these drugs.^[23]^ The International Statistical Classification of Diseases and Related Health Problems (ICD-10) 10th Revision code T42 was used to identify poisoning by antiepileptic, sedative-hypnotic and anti-Parkinsonian drugs hospital admissions.

### 2.3. Ethical considerations

The research ethics committee at the corresponding author’s institution approved the study protocol. Informed consent was obtained from the study participants before the study commencement. This study was conducted under the WMA Declaration of Helsinki.

## 3. Results

### 3.1. Trends in total poisoning by antiepileptic, sedative-hypnotic, and anti-Parkinsonian drugs hospital admissions

Australia recorded a total of 202,705 hospital admission episodes due to poisoning by antiepileptic, sedative-hypnotic, and anti-Parkinsonian drugs between 1998 and 2022. Over this period, the annual number of these hospital admissions for various reasons decreased by 41.7%, dropping from 9723 in 1998 to 5673 in 2022. This decline expressed a reduction in hospital admission rate of 57.8% from 51.68 (95% confidence interval [CI] 50.66–52.71) per 100,000 persons in 1998 to 21.81 (95% CI 21.24–22.37) per 100,000 persons in 2022.

**Figure 1.**
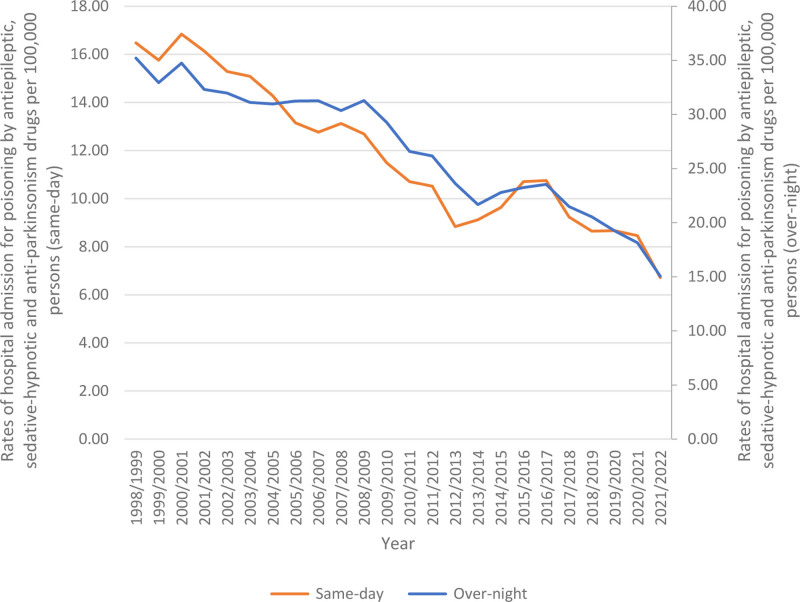
Rates of same-day and overnight-stay patients hospital admission for poisoning by antiepileptic, sedative-hypnotic and anti-parkinsonism drugs in Australia between 1998 and 2022.

Overnight-stay hospital admissions for poisoning by Parkinsonian these drugs accounted for 69.4% of the total, with same-day admissions comprising 30.6%. The rates of same-day hospital admissions Parkinsonian decreased by 59.2%, from 16.47 (95% CI 15.89–17.05) per 100,000 persons in 1998 to 6.72 (95% CI 6.40–7.03) in 2022. Likewise, the rates of overnight-stay hospital admissions decreased by 57.2% from 35.21 (95% CI 34.36–36.06) per 100,000 persons in 1998 to 15.05 (95% CI 14.58–15.52) in 2022 (Fig. 1).

Benzodiazepine poisoning was the most prevalent reason for hospital admission caused by poisoning by antiepileptic, sedative-hypnotic and anti-Parkinsonian drugs, accounting for 76.9% of the total related admissions, followed by poisoning by other antiepileptic and sedative-hypnotic drugs, with 10.9% (Table 1).

**Table 1:** Percentage of poisoning by antiepileptic, sedative-hypnotic and anti-parkinsonism drugs hospital admission from total number of admissions per ICD code.

ICD code	Description	% from total number of admissions
T42.0	Hydantoin derivatives	2.3
T42.1	Iminostilbenes	3.4
T42.2	Succinimides and oxazolidinediones	0.1
T42.3	Barbiturates	0.4
T42.4	Benzodiazepines	76.9
T42.5	Mixed antiepileptics, not elsewhere classified	˂0.1
T42.6	Other antiepileptic and sedative-hypnotic drugs	10.9
T42.7	Antiepileptic and sedative-hypnotic drugs, unspecified	4.0
T42.8	Anti-parkinsonism drugs and other central muscle-tone depressants	2.0

ICD = International Statistical Classification of Diseases system.

### 3.2. Trends in types of poisoning by antiepileptic, sedative-hypnotic and anti-Parkinsonian drugs hospital admissions (separations-based)

**Figure 2.**
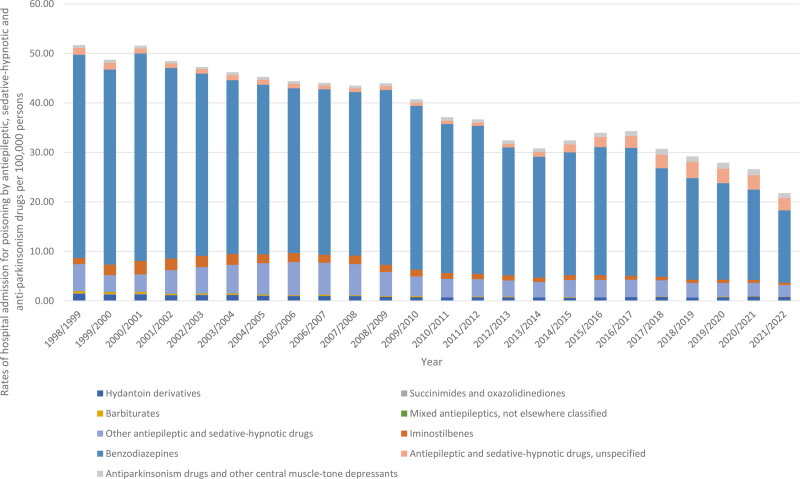
Rates of hospital admission for poisoning by antiepileptic, sedative-hypnotic and anti-parkinsonism drugs in Australia stratified by type between 1998 and 2022.

During the study period, poisoning by “anti-Parkinsonian drugs and other central muscle-tone depressants” and “antiepileptic and sedative-hypnotic drugs, unspecified” increased by 88.3% and 73.2%, respectively. Conversely, poisoning by “mixed antiepileptics, not elsewhere classified,” “succinimides and oxazolidinediones,” “barbiturates,” “benzodiazepines,” “iminostilbenes,” “other antiepileptic and sedative-hypnotic drugs,” and “hydantoin derivatives” decreased by 100%, 100%, 84.2%, 64.4%, 63.4%, 56.2%, and 46.3%, respectively (Table 2, Fig. 2).

**Table 2:** Percentage change in the hospital admission rates for poisoning by antiepileptic, sedative-hypnotic and anti-parkinsonism drugs from 1998 to 2022 in Australia.

Poisonings	Rate of poisonings in 1998 per 100,000 persons (95% CI)	Rate of poisonings in 2022 per 100,000 persons (95% CI)	Percentage change from 1998–2022
Hydantoin derivatives	1.47 (1.29–1.64)	0.79 (0.68–0.90)	−46.3
Iminostilbenes	1.24 (1.08–1.40)	0.45 (0.37–0.54)	−63.4
Succinimides and oxazolidinediones	0.08 0.04–0.12)	0.00 (0.00–0.00)	−100.0
Barbiturates	0.39 (0.30–0.48)	0.06 (0.03–0.09)	−84.2
Benzodiazepines	41.06 (40.14–41.97)	14.60 (14.14–15.07)	−64.4
Mixed antiepileptics, not elsewhere classified	0.04 (0.01–0.06)	0.00 (0.00–0.00)	−100.0
Other antiepileptic and sedative-hypnotic drugs	5.45 (5.12–5.79)	2.39 (2.20–2.57)	−56.2
Antiepileptic and sedative-hypnotic drugs, unspecified	1.42 (1.25–1.60)	2.47 (2.28–2.66)	73.2
Anti-parkinsonism drugs and other central muscle-tone depressants	0.54 (0.43–0.64)	1.01 (0.89–1.13)	88.3

### 3.3. Trends in total poisoning by antiepileptic, sedative-hypnotic, and anti-Parkinsonian drugs hospital admissions (gender-based)

**Figure 3.**
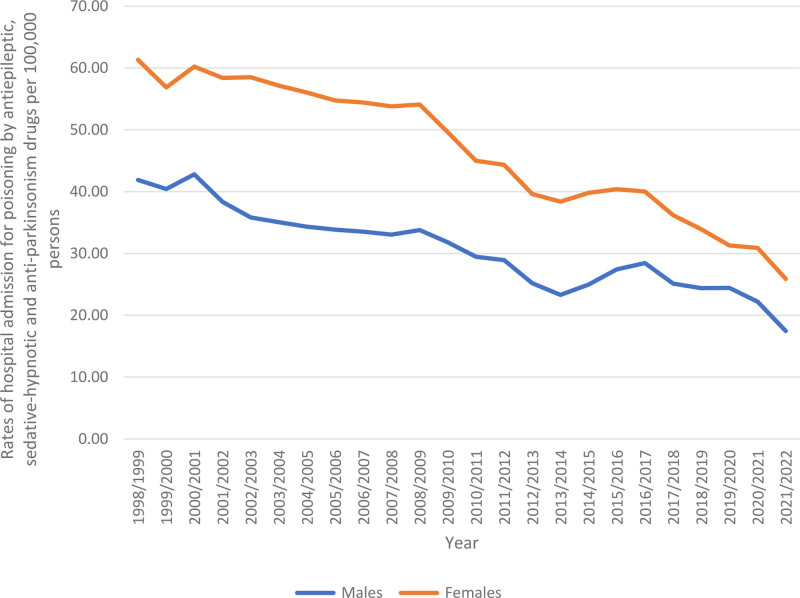
Rates of hospital admission for poisoning by antiepileptic, sedative-hypnotic and anti-parkinsonism drugs in Australia stratified by gender.

Most hospital admissions due to poisoning by antiepileptic, sedative-hypnotic and anti-Parkinsonian drugs (122,777 cases) occurred among females, which represented 60.6% of the total relevant admissions. On average, this equates to 5115.71 patients per year. The hospital admission rate for poisoning by such Parkinsonian drugs among males declined by 58.3% from 41.91 (95% CI 40.59–43.22) per 100,000 persons in 1998 to 17.47 (95% CI 16.75–18.19) in 2022. Similarly, the hospital admission rate for poisoning by these drugs among females declined by 57.8% from 61.33 (95% CI 59.75–62.90) per 100,000 persons in 1998 to 25.90 (95% CI 25.03–26.77) in 2022 (Fig. 3).

### 3.4. Trends in total poisoning by antiepileptic, sedative-hypnotic, and anti-Parkinsonian drugs hospital admissions (age group-based)

**Figure 4.**
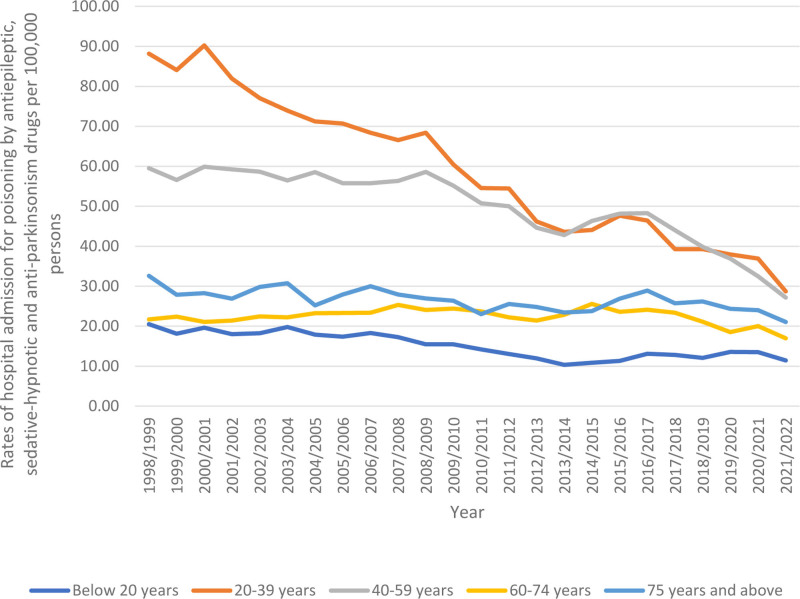
Rates of hospital admission for poisoning by antiepileptic, sedative-hypnotic and anti-parkinsonism drugs in Australia stratified by age group.

In terms of age group distribution for hospital admission due to poisoning by antiepileptic, sedative-hypnotic, and anti-Parkinsonian drugs, the age group 20–39 years accounted for the highest proportion, comprising 43.5% of the total number of cases, followed by 40 to 59 years with 34.2%, below 20 years with 10.2%, 60 to 74 years with 7.6%, and 75 years and above with 4.4%. Furthermore, hospital admission rates for this poisoning Parkinsonian showed a considerable decline across diverse age groups from 1998 to 2022; among patients aged younger than 20 years, the number decreased by 44.3% (from 20.51 (95% CI 19.28–21.73) in 1998 to 11.42 (95% CI 10.58–12.25) in 2022 per 100,000 people). For patients aged 20 to 39 years, rates decreased by 67.5% (from 88.18 (95% CI 85.73–90.63) in 1998 to 28.70 (95% CI 27.47–29.93) in 2022 per 100,000 people). Among patients aged 40 to 59 years, the rates declined by 54.4% (from 59.53 (95% CI 57.36–61.70) in 1998 to 27.16 (95% CI 25.90–28.43) in 2022 per 100,000 people). For patients aged 60 to 74 years, rates fell by 21.8% (from 21.70 [95% CI 19.69–23.71] in 1998 to 16.98 [95% CI 15.69–18.26] in 2022 per 100,000 people). Among patients aged 75 years and older, rates decreased by 35.4% (from 32.60 [95% CI 29.10–36.10] in 1998 to 21.05 [95% CI 19.03–23.07] in 2022 per 100,000 people) (Fig. 4).

### 3.5. Trends in poisoning by antiepileptic, sedative-hypnotic and anti-Parkinsonian drugs hospital admissions (gender-based)

**Figure 5.**
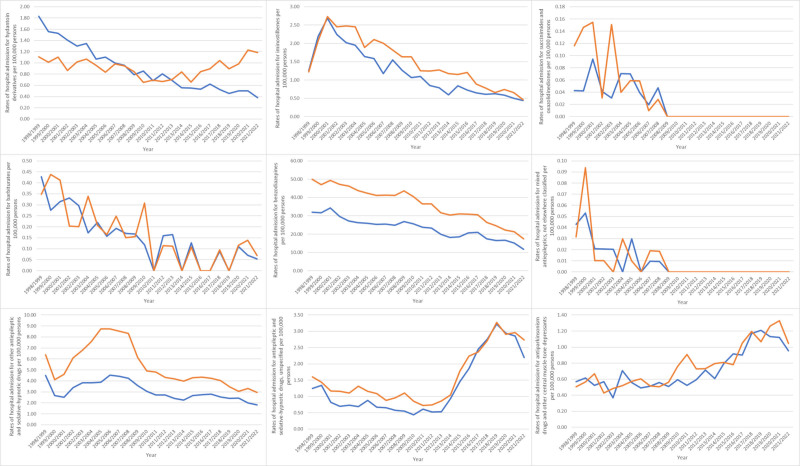
Rates of hospital admission for poisoning by antiepileptic, sedative-hypnotic and anti-parkinsonism drugs in Australia stratified by gender.

Throughout the study, hospital admission rates for poisoning by antiepileptic, sedative-hypnotic and anti-Parkinsonian drugs were greater among females in comparison to males (Fig. 5).

### 3.6. Trends in poisoning by antiepileptic, sedative-hypnotic, and anti-Parkinsonian drugs hospital admissions (age group-based)

**Figure 6.**
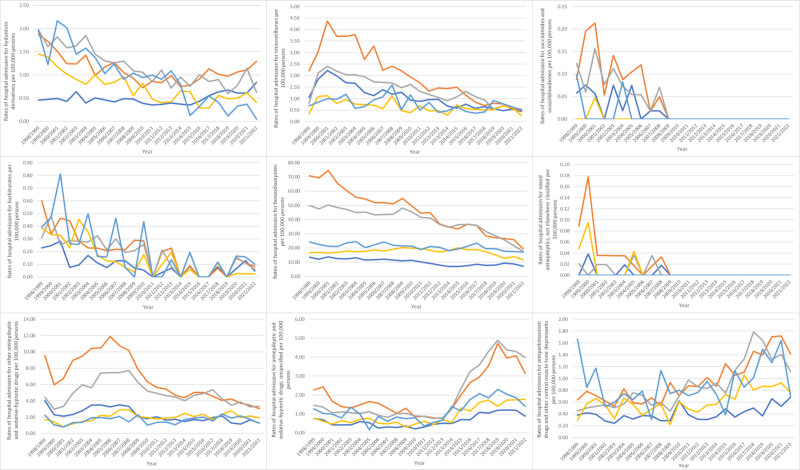
Rates of hospital admission for poisoning by antiepileptic, sedative-hypnotic and anti-parkinsonism drugs in Australia stratified by age group.

Most hospital admission rates for poisoning by antiepileptic, sedative-hypnotic and anti-Parkinsonian drugs were more common in the 20 to 39 years age group. That encompassed iminostilbenes, “succinimides and oxazolidinediones,” benzodiazepines, “mixed antiepileptics, not elsewhere classified,” “other antiepileptic and sedative-hypnotic drugs,” “antiepileptic and sedative-hypnotic drugs, unspecified,” and “anti-Parkinsonian drugs and other central muscle-tone depressants.” However, hospital admission rates for poisoning by hydantoin derivatives and barbiturates were more prevalent respectively among the 40 to 59 and 75 years and above age groups (Fig. 6).

## 4. Discussion

Poisoning negatively impacts the patient’s and family’s quality of life and societal, spiritual, emotional, and physical well-being.^[24,25]^ Our study highlights the trend in hospital admissions due to poisoning with antiepileptic, sedative-hypnotic, and anti-Parkinsonian drugs in Australia. Our analysis has revealed several important patterns that require attention from public health experts, policymakers, and healthcare workers.

The results of this study found a considerable reduction in the total annual number of hospital admissions for diverse causes of poisoning by antiepileptic, sedative-hypnotic and anti-Parkinsonian drugs by 41.7% from 1998 to 2022, with a notable 57.8% decline in the related hospital admission rate over the same period. By comparison, previous research conducted in England, Wales, and the Lower Silesia region reveals differences and similarities. For example, earlier studies in England and Wales reported only a 0.1% decline in the total annual number of hospital admissions related to such poisoning Parkinsonian from 1999 to 2020, with a 12.8% reduction in hospital admission rates.^[26]^ On the other hand, in the Lower Silesia region, a prior study indicated unstable hospital admissions trends related to poisoning with these drugs between 2006 and 2012, with an overall increasing trend.^[12]^ It should be noted that multiple factors can contribute to discrepancies between studies, such as regional differences in healthcare practices, drug distribution patterns, and the effectiveness of health strategies.

Factors that may have resulted in the reduction in hospital admissions related to poisonings by Parkinsonian these drugs in our study may include changes in the dispensing rate of these drugs and the implementation of healthcare system interventions. For example, benzodiazepine dispensing rates decreased significantly in Australia from 6582,561 in 2012 to 2013 to 4891,862 in 2021 to 2022.^[17]^ Furthermore, a prior report underlined positive outcomes associated with the Australian National Drug Control Strategy,^[27]^ which could have contributed to this decline. These emphasize the importance of public health interventions and awareness campaigns in diminishing drug-related harm and hospital admissions.

Our study has demonstrated that overnight stays accounted for the majority of hospital admissions for patients admitted due to this poisoning, Parkinsonian amounting to 69.4% of the total. This implies that most patients required medical intervention, intensive care, or extended monitoring.

In our research, we observed that benzodiazepine poisoning was the foremost cause of hospital admissions associated with this poisoning, Parkinsonian accounting for 76.9% of all such admissions, followed by poisoning by other antiepileptic and sedative-hypnotic drugs, accounting for 10.9% of cases. Moreover, we observed an increase in hospital admissions related to poisoning by “anti-Parkinsonian drugs and other central muscle-tone depressants” by 88.3% and poisoning by “antiepileptic and sedative-hypnotic drugs, unspecified” by 73.2%.

Regarding the leading cause of hospital admissions related to poisoning by antiepileptic, sedative-hypnotic and anti-Parkinsonian drugs, these results align with numerous findings from previous research. A prior study conducted in England and Wales revealed that poisoning with benzodiazepines and other antiepileptic and sedative-hypnotic drugs was the leading cause of hospital admission related to poisoning by antiepileptic, sedative-hypnotic and anti-Parkinsonian drugs, which accounted for 54.2% and 30.7% of all related admissions, respectively.^[26]^ A previous article has studied the toxicity of anxiolytics and sedative-hypnotic drugs and documented that abuse of benzodiazepine drugs was more prevalent than other drugs.^[28]^ As mentioned earlier, benzodiazepines accounted for about 46% of hospital admissions related to antiepileptic, sedative-hypnotic and anti-Parkinsonian drugs in Australia,^[17]^ and 54% of fatalities related to drug overdose were due to such drugs Parkinsonian.^[11]^ Notably, benzodiazepines were the principal drugs, accounting for 45% of these fatalities. In our study, benzodiazepine-related hospital admissions decreased by 64.4%, indicating the effectiveness of the implemented strategies in Australia, as discussed previously. However, the overlap between hospital admission and mortality data emphasizes that there is still a need to address benzodiazepine abuse.

Understanding the risk factors associated with poisoning by sedatives (like female gender, smoking, alcohol addiction or abuse, lack of insurance, white race, the existence of panic manifestations, unemployment, and others^[29]^) can help in the development of preventive strategies and interventions to reduce poisonings and hospital admissions associated with the use of such sedatives.

The results of previous studies highlight several factors that are in line with the results observed in our study. First, the complex pharmacokinetic properties, instabilities in drug concentration, and narrow therapeutic index of antiepileptic drugs underscore the potential toxicity risks,^[30]^ consistent with the observed increase in hospital admissions for poisoning by “antiepileptic and sedative-hypnotic drugs, unspecified.” Moreover, the heightened utilization of sedative-hypnotics in diverse settings,^[28]^ in addition to the high incidence of mood disorders and neurodegenerative diseases such as Parkinson disease,^[31,32]^ further results in the rising number of cases related to sedative-hypnotics, anti-Parkinsonian, and other centrally acting muscle-tone depressant drugs. The aging demographics in Australia, besides the expected rise in Parkinson disease cases, create noteworthy public health burdens.^[33–37]^ These predictions are consistent with the increase in hospital admissions due to poisoning by antiparkinsonian drugs observed in our study. These findings indicate a potential complex association between hospital admissions due to poisoning and drug properties, drug usage trends, demographic shifts, disease prevalence, and public health challenges, and underscore the necessity for conducting future interventions to alleviate the risks associated with poisoning by antiepileptic, sedative-hypnotic, and anti-Parkinsonian drugs.

Our study highlights the significant burden of poisoning by antiepileptic, sedative-hypnotic and anti-Parkinsonian drugs among females. This demonstrates that females represent a considerable proportion of hospital admissions due to poisoning by these drugs, as they comprise 60.6% of the total cases observed during the study period. These findings are consistent with previous research conducted in England and Wales, where 55.5% of the total hospitalizations related to these drugs occur among females.^[26]^ Moreover, a prior study emphasized the heightened hospital admissions rates of self-harm poisoning among females.^[38]^ All these findings underline the essence of understanding and addressing the factors contributing to this trend among female patients. As previously mentioned, female gender is a risk factor associated with poisoning by sedatives.^[28]^ Understanding the reasons for the heightened hospital admissions due to poisoning by antiepileptic, sedative-hypnotic and anti-Parkinsonian drugs among females is essential for developing effective and preventive interventions, especially for this demographic group.

Our study found that individuals aged 20 to 39 years accounted for the most significant proportion of hospital admissions related to poisoning by antiepileptic, sedative-hypnotic and anti-Parkinsonian drugs at 43.5%, followed by those aged 40 to 59 years at 34.2%. That nearly aligned with findings from a previous report in Australia, which underlined higher hospital admission rates among young to middle-aged adults, particularly in the 20 to 29 and 30 to 39 age groups.^[39]^ Moreover, a prior study in Australia found that hospitalizations due to psychotropic substance administration errors were more common among the age groups 20 to 29 and 40 to 59 years.^[40]^ Likewise, research in England and Wales has identified that the 15 to 59 age group accounts for approximately 85% of hospital admissions for poisoning by these drugs.^[26]^ A trend in poisoning by antiepileptic, sedative-hypnotic and anti-Parkinsonian drugs-related hospital admissions among young and middle-aged adults underscores the global nature of this demographic trend across different regions. These findings emphasize the need to consider age groups when developing strategies to reduce hospital admissions due to poisoning. Effective interventions in the management of antiepileptic, sedative-hypnotic and anti-Parkinsonian drugs-related poisoning and admissions are essential to enhance treatment safety in this population.

This study has limitations. The ecological study design’s ability to evaluate population trends rather than individuals’ risk makes it subject to ecological fallacy. Besides, we were unable to obtain further medical information related to patients’ comorbidity, which restricted the ability to identify important confounding variables. We were also unable to establish a causal relationship between changes in benzodiazepine dispensing, national drug policies, and observed trends in hospital admissions because our study did not include a direct analysis of the medication prescriptions profile. Consequently, caution should be exercised when interpreting the associations proposed in this study and should be regarded as hypothesis-generating rather than conclusive. Moreover, assessing the actual clinical severity was restricted by the absence of data on ICU admissions. Therefore, our findings may have underestimated or overestimated the actual burden on the health system by concentrating solely on admission frequency.

Future research is recommended to incorporate stochastic modeling approaches. They are valuable mathematical frameworks for modeling complex, dynamic systems under uncertainty.^[41,42]^ The application of stochastic differential equations to adverse drug reaction-related hospital admissions would enable the simulation of a variety of intervention strategies, capture system variability, and provide more precise predictions of future admission rates. These methodologies serve as a prospective avenue for the advancement of methodological rigor in this field by facilitating the creation of predictive tools that integrate both probabilistic fluctuations and deterministic trends, which, in turn, complement the current findings.^[41,42]^

## 5. Conclusion

During the study period, hospital admissions for antiepileptic, sedative-hypnotic, and anti-Parkinsonian drug poisoning declined significantly in Australia. The decline in hospital admissions for poisonings for most classes of drugs studied reflects the effectiveness of drug regulation strategies and public health interventions. Despite this, hospital admission rates increased for poisoning by “anti-Parkinsonian drugs and other central muscle-tone depressants” and “antiepileptic and sedative-hypnotic drugs, unspecified.” The female population and the 20 to 39 age group were at higher risk of hospital admission. These findings suggest the necessity for continuous monitoring, targeted interventions, and improved understanding among the population to further diminish drug poisoning and related hospital admissions.

## Acknowledgments

We would like to acknowledge Princess Nourah bint Abdulrahman University Researchers Supporting Project number (PNURSP2025R483), Princess Nourah bint Abdulrahman University, Riyadh, Saudi Arabia.

## Author contributions

**Conceptualization:** Abdallah Y. Naser.

**Data curation:** Abdallah Y. Naser.

**Formal analysis:** Abdallah Y. Naser.

**Funding acquisition:** Alaa A. Alsharif.

**Investigation:** Abdallah Y. Naser, Hassan Alwafi, Alaa A. Alsharif, Ahmed M. Al Rajeh, Jaber S. Alqahtani, Abdulelah M. Aldhahir, Anan S. Jarab, Yosra J. Alhartani, Asaleh El-Qasem.

**Methodology:** Abdallah Y. Naser.

**Project administration:** Abdallah Y. Naser, Alaa A. Alsharif, Ahmed M. Al Rajeh.

**Resources:** Abdallah Y. Naser, Hassan Alwafi, Alaa A. Alsharif, Ahmed M. Al Rajeh, Jaber S. Alqahtani, Abdulelah M. Aldhahir, Anan S. Jarab, Yosra J. Alhartani, Asaleh El-Qasem.

**Software:** Abdallah Y. Naser.

**Supervision:** Abdallah Y. Naser.

**Validation:** Abdallah Y. Naser, Alaa A. Alsharif.

**Visualization:** Abdallah Y. Naser.

**Writing – original draft:** Abdallah Y. Naser, Ahmed M. Al Rajeh.

**Writing – review & editing:** Abdallah Y. Naser, Hassan Alwafi, Alaa A. Alsharif, Ahmed M. Al Rajeh, Jaber S. Alqahtani, Abdulelah M. Aldhahir, Anan S. Jarab, Yosra J. Alhartani, Asaleh El-Qasem.
